# Unraveling Concussion Impact by Impact 

**DOI:** 10.16910/jemr.12.7.5

**Published:** 2019-11-25

**Authors:** Janet C. Rucker

**Affiliations:** Langone, New York University

**Keywords:** concussion, eye movements

## Abstract

Keynote at the 20th European Conference on Eye Movement Research (ECEM) in Alicante, 20.8.2019.

**Video stream:** https://vimeo.com/357889739

